# 2248: Screening for diabetes in high-risk women: Building the data infrastructure to study postpartum diabetes screening among low-income women with gestational diabetes

**DOI:** 10.1017/cts.2017.254

**Published:** 2018-05-10

**Authors:** Cynthia Joan Herrick, Ben Cooper, Matthew Keller, Margaret Olsen, Graham Colditz

**Affiliations:** Institute of Clinical and Translational Sciences, Washington University in St. Louis, St. Louis, MO, USA

## Abstract

OBJECTIVES/SPECIFIC AIMS: Women with GDM have a 7-fold higher risk of developing T2DM, and rates of GDM are higher among racial and ethnic minorities and women of lower socio-economic status. There are no data on postpartum diabetes screening after the first postpartum year or among women receiving care in FQHCs. We aim to address this gap in the literature by (1) defining the rates of follow-up screening for T2DM at 6–12 weeks and 1–3 years postpartum and (2) characterizing patient, provider, and healthcare system attributes that are associated with lack of follow-up screening for T2DM in a population of low-income women with GDM. METHODS/STUDY POPULATION: This is a retrospective cohort study of women with GDM during pregnancy receiving care in Missouri FQHCs from 2010 to 2015. Electronic health records (EHR) data from 26 FQHCs is housed in a central repository through the Missouri Primary Care Association (MPCA). This includes patient demographic, lab, and medication information as well as encounter level patient and provider data for the prenatal and postpartum period. EHR data does not include accurate delivery information, however. Pregnancies during the study time frame were identified using CPT and ICD9/10 codes. Deidentified data on individuals with a pregnancy was utilized to identify a subpopulation of “GDM candidates,” using a broad definition of glucose abnormalities as follows: ICD-9/ICD-10 codes for diabetes, medications and testing supplies used for diabetes, infant birth weight ≥4000 g or 8 lb or 13 oz, or abnormal glucose labs [defined as fasting glucose≥95, gestational glucose screen≥130, 1 h test≥130 (or ≥180 if 2 h test and 3 h test recorded on same day), 2 h test≥155, 3 h test≥140, A1C≥6, any glucose≥130, or any positive urine glucose]. This subpopulation was then linked to Medicaid administrative claims data [housed at the University of Missouri Office of Social and Economic Development Analysis (OSEDA)], providing detailed information on delivery, to further characterize patients with GDM in the time frame and provide all dates necessary to classify pregnancy and postpartum periods. RESULTS/ANTICIPATED RESULTS: From the de-identified pregnancy data set including 45,810 individuals, we identified 8008 “GDM candidates.” EHR data were linked to Medicaid claims data for these individuals from 2010 to 2015. Utilizing the enhanced data set, we are defining a pregnancy for each individual by the delivery date in the Medicaid record and an algorithm using lab and ultrasound record dates to define gestational age at delivery. This will result in a pregnancy level data set linked with individual encrypted identifiers with each record representing 1 pregnancy and postpartum period. GDM in pregnancy will be defined as having a baby with birth weight≥4000 g or 8 lb or 13 oz, ICD-9 or ICD-10 code for GDM during pregnancy or at delivery, or an oral glucose tolerance test (oGTT) 12–16 weeks before delivery with 2 or more abnormal results by Carpenter and Coustan criteria. We anticipate that our final GDM data set will include 2000–3000 individuals. We will then calculate the percentage of individuals receiving recommended screening tests at 6–12 weeks (fasting glucose or 2 h oGTT) and 1–3 years postpartum (fasting glucose, 2 h oGTT, HbA1C). We will use multivariable regression techniques to identify risk factors for lack of screening. We will be able to incorporate predictors not previously evaluated including distance from home to health center, access to public transport, specialty and training of the patient’s providers, pregnancy weight gain, postpartum appointment time of day, and number of various types of office visits. DISCUSSION/SIGNIFICANCE OF IMPACT: The creation of a linked data set of pregnancies complicated by GDM in women receiving care in FQHCs in Missouri is the first step toward better characterizing follow-up diabetes screening rates in this population and understanding patient, provider, and healthcare system variables that affect postpartum screening. The ultimate goal is to translate evidence-based patient-centered sustainable interventions into practice for low-income women with a history of GDM and improve population outcomes with the ability to track progress prospectively over time.

Acknowledgements: The authors thank Susan Wilson (MPCA), Jill Lucht, and Bhawani Mishra (OSEDA).

